# Comparative Outer Membrane Protein Analysis of High and Low-Invasive Strains of *Cronobacter malonaticus*

**DOI:** 10.3389/fmicb.2017.02268

**Published:** 2017-11-17

**Authors:** Maha A. Aldubyan, Ibtesam S. Almami, Fatiha M. Benslimane, Abdlrhman M. Alsonosi, Stephen J. Forsythe

**Affiliations:** ^1^School of Science and Technology, Nottingham Trent University, Nottingham, United Kingdom; ^2^Department of Pharmacology, College of Pharmacy, Qassim University, Al-Qassim, Saudi Arabia; ^3^Biology Department, College of Science, Qassim University, Al-Qassim, Saudi Arabia; ^4^Biomedical Research Institute, Qatar University, Doha, Qatar

**Keywords:** *Cronobacter* spp., *Cronobacter malonaticus*, invasive, outer membrane proteins (OMPs), proteomics, flagellin

## Abstract

*Cronobacter* are an important group of foodborne pathogens that has been linked to life-threatening infections in both infants and adults. The major infections associated with *Cronobacter* species are neonatal meningitis, necrotizing enterocolitis, and septicaemia. There are seven species in the *Cronobacter* genus, of which only three are of clinical importance; *Cronobacter sakazakii, Cronobacter malonaticus*, and *Cronobacter turicensis*. To date most studies have focussed on *C. sakazakii* as it is the major species associated with neonatal infections. However, recently *C. malonaticus*, in particular sequence type 7 (ST7), has been noted as being prevalent in adult infections and therefore warranting further investigation. In this study, eight strains of *C. malonaticus* ST7, that had been isolated from a wide range of sources and varied in their *in vitro* virulence, were chosen for proteomic analysis of their outer membrane proteins (OMPs). One-dimensional gel analysis revealed a ~29 kDa size band that was only present in the highly invasive strains. Subsequent mass spectrometric analysis identified several peptides that matched the flagellin protein. The presence of flagellin protein was confirmed in 2D gel spot. Mass spectrometry analysis of total OMPs revealed that the four highly invasive *C. malonaticus* strains expressed the main flagellum proteins that were absent from the four low invasive strains. These were the flagellar hook protein FlgE, flagellar hook-associated protein 1, flagellar hook-associated protein, flagellin, and flagellar hook-filament junction protein FlgL. This data indicates that *C. malonaticus* flagellar proteins may have an important role in the organism's invasion properties.

## Introduction

*Cronobacter* species are Gram-negative, facultative anaerobic rod-shaped bacteria and are members of the Enterobacteriaceae family (Iversen et al., 2008). The genus is closely related to the *Enterobacter* and *Citrobacter* genera (Forsythe et al., 2014). Seven *Cronobacter* species have been identified to date; *Cronobacter sakazakii, Cronobacter malonaticus, Cronobacter turicensis, Cronobacter universalis, Cronobacter muytjensii, Cronobacter dublinensis*, and *Cronobacter condimenti* (Cetinkaya et al., 2013). They occur in the environment, plant material and subsequently the ingredients of various foods (Forsythe et al., 2014). *Cronobacter* species have been isolated from a wide range of sources including food, rats, flies, and asymptomatic human carriage has been reported (Kandhai et al., 2004; Friedemann, 2007; Miled-Bennour et al., 2010). The organism has been isolated from the hospital environment and clinical samples, including cerebrospinal fluid, blood, bone marrow, sputum, urine, inflamed appendix, neonatal enteral feeding tubes, and conjunctivae (Iversen and Forsythe, 2003; Hurrell et al., 2009; Holy and Forsythe, 2014).

*Cronobacter* species are opportunistic pathogens associated with severe neonatal infections; septicaemia, necrotizing enterocolitis, and meningitis (Biering et al., 1989; Joseph and Forsythe, 2011; Holy and Forsythe, 2014). Clinical cases of *Cronobacter* infection have been reported in all age groups, with the majority being in the adult population (Lai, 2001; Holy et al., 2014; Patrick et al., 2014; Alsonosi et al., 2015). Although infections in infants and neonates are rare, they can have fatal consequences (Caubilla-Barron et al., 2007). The majority of infant and neonatal cases, including outbreaks on neonatal intensive care units, are only associated with the *C. sakazakii* species (Kucerova et al., 2011; Forsythe et al., 2014), especially the neonatal meningitic pathovar *C. sakazakii* sequence type 4 (ST4; Joseph and Forsythe, 2011; Hariri et al., 2013). This could be linked to their frequent isolation from infant formula and milk powder manufacturing plants, where 25% of isolates have been reported to be this pathovar (Sonbol et al., 2013).

Clinical isolates of *C. sakazakii* are able to adhere and invade intestinal cells and brain cells as well as survive in macrophages (Townsend et al., 2007, 2008; Giri et al., 2012). The organism is able to invade and translocate through intestinal (Caco-2) and brain (HBMEC) cell lines as well as persist and multiply within macrophages and microglial cells (Almajed and Forsythe, 2016). Due to the association of *C. sakazakii* with infant and neonatal outbreaks, the majority of virulence-related studies have focused only on this species. Consequently, few studies have considered the virulence traits of *C. malonaticus*, which is associated with adult infections and thus the majority of *Cronobacter* infections (Forsythe et al., 2014).

This study analyzed the outer membrane proteins (OMPs) of eight ST7 *C. malonaticus* strains, which had been isolated from a wide range of sources and periods. Furthermore, the strains varied in their virulence based on *in vitro* studies (Alsonosi, 2017).

## Materials and methods

### Bacterial strains and growth conditions

Eight *C. malonaticus* strains (Table 1) were recovered from storage at −80°C by streaking on Tryptone Soya agar (TSA) and incubating for 18 h at 37°C. The strains were then cultured in 400 ml of Tryptone Soya broth (TSB) (CM0131, Oxoid Thermo Fisher) for 18 h with gentle shaking at 37°C.

**Table 1:** *Cronobacter malonaticus* strains used in this study.

**Strain number**	**Sequence type**	**Source**	**Country**	**Year of isolation**	***In vitro* invasion ability**
565^a^	7	Fecal isolate	USA	1973	High
1558^b^	7	Fecal isolate	Czech Republic	1980	High
1827^c^	7	Cannula (Blood)	Czech Republic	2007	High
2018^c^	7	Sputum	Czech Republic	2013	High
1830^c^	7	Throat swab	Czech Republic	2007	Low
1833^c^	7	Faecal isolate	Czech Republic	2010	Low
1835^c^	7	Throat swab	Czech Republic	2012	Low
2020^c^	7	Fecal isolate	Czech Republic	2013	Low

a *Farmer et al. (1980)*.

b *Aldova et al. (1983)*.

c *Alsonosi et al. (2015)*.

### Outer membrane isolation and protein estimation

Three 400 ml cultures of each *C. malonaticus* strain were grown then pelleted by centrifugation at 5,000 × g for 25 min. The pellets were then washed twice with PBS and re-suspended in 5 ml 10 mM HEPES (Sigma, UK), pH 7.5 with 1 μl/ml protease inhibitor and lysed using a sonicating water bath in ice for 7 min. The membranes were collected by ultracentrifugation (Beckman-Coulter Centrifuge Optima L100XP) at 100,000 × *g* for 1 h at 4°C. The pellets were re-suspended in 5 ml of 10 mM HEPES, pH 7.5 with 1 μl/ml protease inhibitor, using 20-gauge needle, and spun using ultracentrifugation as describe above. The pellet was re-suspended in 4 ml of 1% (w/v) *N-*lauroylsarcosine (Sigma, UK) in 10 mM HEPES, pH 7.5 with 1 μl/ml protease inhibitor, and incubated at 37°C for 30 min with shaking and spun again in ultracentrifugation as describe above. The *N-*lauroylsarcosine-treated membranes were spun at 100,000 × *g* for 1 h at 4°C and the pellet washed briefly with 1 ml of 10mM HEPES, pH 7.5 with 1 μl/ml protease inhibitor. The pellet was re-suspended in 400 μl of 10mM HEPES, pH 7.5 with 1 μl/ml protease inhibitor. The concentration of isolated OMPs were measured by the bicinchoninic acid (BCA) protein assay, based on the method of (Stoscheck, 1990) using a kit from Sigma (Poole, UK). The absorbance of the samples was measured at 570 nm using a plate reader (Expert 96, Scientific laboratory, UK) and a calibration graph was plotted for protein range 0–1 mg ml^−1^.

### Sodium dodecylsulphate-polyacrylamide gel electrophoresis (SDS-PAGE)

The isolated OMPs were separated on 10% sodium dodecylsulphate-polyacrylamide gel electrophoresis using the method described by Laemmli (1970). Any bubbles generated during preparation were removed by overlaying with isopropanol. Briefly, 50 μg OMPs were mixed with 5 μl of 6x SDS-sample buffer and boiled for 5 min. After cooling, the denatured samples were loaded in 10% SDS-gel wells along with 3 μl of molecular weight Precision Plus Protein standards (Bio-Rad, UK). Using the Bio-Rad (Bio-Rad Laboratories, USA) apparatus, the gel was subjected to electrophoresis at 175 V for 1 h. The resolved OMPs were then visualized using InstantBlue Protein Stain (Sigma-Aldrich, UK) flowed by destaining with water.

### Two-dimensional gel electrophoresis

Two-dimensional gel electrophoresis was as according to Nirmalan et al. (2004). The OMPs were acetone precipitated, and the protein pellet was dissolved in rehydration buffer containing; 8 M urea, 4% (w/v) CHAPS, 50 mM DTT, 0.2% (v/v) carrier ampholytes, 0.0002% (w/v) Bromophenol Blue. In the first-dimension isoelectric focusing, an equal amount of protein was loaded on immobilized pH gradient (IPG) strips with a pH range of 3–10 and incubated for 2 h at room temperature. The gel strips were then focused with a Protean isoelectric focusing cell (Bio-Rad Laboratories, Hercules, USA) and the program was set as follows: rehydrating for 16 h at 50 V at 20°C, focusing at 250 V for 15 min, followed by 8,000 V for 2 h. For the second-dimension separation, the IPG strips were equilibrated for 15 min with 10% (w/v) DTT in 10 ml of an equilibration buffer [50 mM Tris base, pH 8.8, 6 M urea, 30 % (v/v) glycerol, and 2% (w/v) SDS] and for further 15 min with 25% (w/v) iodoacetamide in the equilibration buffer. Each IPG strip was loaded onto a gel of the appropriate percentage of acrylamide, sealed with 1% (w/v) agarose, and 10% polyacrylamide gel electrophoresis was performed as describe above. After electrophoresis, the proteins were fixed and visualized using PlusOne silver staining kit (GE Healthcare life science, UK) according to the manufacturer's suggested protocol then visualized with Biomolecular Imagers (FLA 7000; Life Sciences, UK). For spot intensity comparison, isolated OMPs from three independent replicates were analyzed by Progenesis SameSpots software, UK.

### Maldi-TOF/TOF and LC-MS/MS analysis

#### In gel digestion

The protein bands were excised from either the 10% polyacrylamide gel or the 2D gel electrophoresis second-dimension gel. For in gel digestion, the gel bands or spots were excised and cut into 1 mm pieces. Gel pieces were washed twice with >10 volumes of Millipore water (~200 μl) for 30 s, to wash out any acetic acid. Gel pieces were destained with freshly prepared 200 μl of 100% methanol and 50 mM (NH_4_)HCO_3_ (1:1 v/v) for 1 min and then dehydrated for 5 min at room temperature with 200 μl of 100% (v/v) acetonitrile in 50 mM (NH_4_)HCO_3_ (1:1 v/v) with vortex. The dehydration solution was removed and 100% (v/v) acetonitrile was added and incubated for 30 s or until the gel pieces shrunk. Then the buffer was removed and the gel pieces were left to air dry. For reduction and alkylation, gel pieces were rehydrated with 100 μl of freshly prepared 25 mM DTT (Sigma, UK) in 50 mM (NH_4_)HCO_3_ and were incubated for 20 min at 56°C. Buffer was removed and replaced with 100 μl of freshly prepared 55 mM iodoacetamide (Sigma, UK) in 50 mM (NH_4_)HCO_3_ solution and incubated in the dark for 15 min at 25°C. The gel pieces were once more dehydrated by 200 μl of 100% (v/v) acetonitrile in 50 mM (NH_4_)HCO_3_ (1:1 v/v) with vortex. The dehydration solution was removed and 100% (v/v) acetonitrile was added and mixed for 30 s until the gel pieces shrank. Acetonitrile was then removed and the gel pieces were dried by removing excess liquid and left to air dry.

#### Trypsin digestion

Proteins in the gel pieces were digested with trypsin overnight at 37°C in 20 μl of digestion solution containing 12 ng/μl trypsin gold (Promega, UK) in 0.01% ProteaseMAX™ Surfactant:50 mM (NH_4_)HCO_3_. The reaction was terminated by adding 0.5% (v/v) trifluroacetic acid (TFA) to a final concentration of 0.5% (v/v) then mixing for 5 min. Digested proteins were recovered by centrifugation for 10 min at maximum speed ~8,000 × g (Scientific Laboratory, UK). The supernatant containing the digestion solution was transferred to a new tube. The sample was then ready for LC-based mass analysis.

#### In solution digestion with trypsin

From all OMP extracts, 50 μg of proteins were precipitated in acetone. The protein pellets were dissolved in solubilization buffer containing 0.2% ProteaseMAX™ surfactant in 50 mM (NH_4_)HCO_3_ (1:1 v/v). The protein pellets were then incubated with solubilization buffer in an orbital shaker at 140 rpm for 10 min. A final volume of 93.5 μl was reached by adding 50mM (NH_4_)HCO_3_. The protein samples were subsequently incubated with 1 μl of 0.5 M DTT in 50 mM (NH_4_)HCO_3_ at 56°C for 20 min. Afterwards, 2.7 μl of 55 mM iodoacetamide in 50 mM (NH_4_)HCO_3_ was added and sample tubes were incubated in the dark at 25°C for 15 min. A total of 1 μl of 1% ProteaseMAX™ Surfactant and 1.8 μl of 1 μg/μl trypsin were then added and the protein samples were incubated overnight at 37°C. The reaction was terminated by adding 0.5% (v/v) TFA and mixed for 5 min, this raised the final volume to 100 μl. Digested proteins were recovered by centrifugation for 10 min at max speed ~8,000 × g (Scientific Laboratory, UK).

#### Peptide purification and proteomic analysis

Digested peptides from 2D gel spots were purified using ZipTip-C_18_ columns (Millipore, UK) which contained 0.5 μl of immobilized chromatography media (C18, resin) at their distal end for sample clean-up before spotting onto a MALDI plate. The Zip-Tip was washed five times with 0.1% (v/v) TFA in acetonitrile, followed with five times wash with 0.1% (v/v) TFA in 1:1 acetonitrile: water. The Zip-Tip was equilibrated twice with 0.1% (v/v) TFA in water and the digested peptides were passed through the Zip-Tips repeatedly by pipetting in and out to bind the sample to the resin. This was followed by washing three times with 0.1% TFA and 5% (v/v) methanol in water to remove unbound material. The sample was eluted directly from the Zip-Tip in 3 μl of 80% (v/v) acetonitrile through 15 aspirating and dispensing cycles. A 1 μl volume of eluted sample was mixed with 1.1 μl of matrix, typically alpha-cyano-4-hydroxycinnamic acid in 0.1% (v/v) TFA 80% (v/v) acetonitrile and spotted in triplicate on the MALDI-TOF sample plate and were analyzed using a MALDI-TOF-MS (UltrafleXtreme, Bruker Daltonics, Germany).

Peptide Mass Fingerprint (Mascot) was searched assuming the digestion enzyme trypsin, Mass values was Monoisotopic, Peptide Mass Tolerance; ± 100 ppm, Peptide Charge State; 1+, Max Missed Cleavages; 1 variable modification; Oxidation (M) and fixed was Carbamidomethyl (C). Data was search at NCBIprot and taxonomy was defined for Proteobacteria (Cottrell, 2011).

The full OMP protein profiling was on LC-MS/MS (Eksegent 400 LC hyphenated to the SCIEX 5600 triple-TOF, USA), LC-fractions were controlled using WARP-LC software (version 3.2, Bruker Daltonics) and FlexControl software (version 3.3, Bruker Daltonics). A total of either 1 μl or a maximum load of 3 μl (0.5 μl or 1.5μg, respectively) of the digested proteins was injected and the data acquired were searched against a *C. sakazakii* database in SWISSPROT using MASCOT (version 2.3 servers, Matrix Science). Data generated from the 3 μl injection gave better protein query converge and as such was used for the comparative analysis.

### Motility test

Twenty grams of Luria Bertani (LB) broth (Sigma Aldrich, UK) and 0.4% of agar technical (Thermo Fisher Scientific, UK) were dissolved in 1,000 ml of distilled water and then 5 ml of 1% triphenyl-tetrazolium chloride (TTC) solution (Fluka, UK) was added. The mixture was autoclaved at 121°C under 15 psi pressure for 15 min. After autoclaving, the media was dispensed aseptically into Petri dishes. One colony of fresh TSA culture was inoculated into 5 ml of TSB and incubated in a shaking incubator at 37°C for 18 h. Three microliters of each overnight culture broth was inoculated into the center of the TTC-LB agar and incubated overnight at 37°C. *Salmonella* Enteritidis and *Klebsiella pneumoniae* were used as positive and negative motility controls, respectively.

## Results

**Figure 1:**
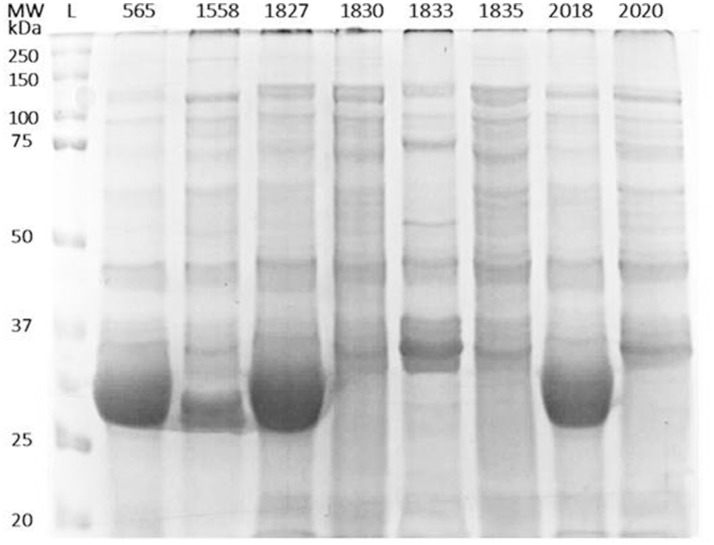
Outer-membrane proteins profile of the eight *C. malonaticus* strains. Cell pellets from 400 ml of culture of *C. malonaticus* strains were subjected to subcellular fractionation by ultracentrifugation using 1% (w/v) N-lauroylsarcosine in 10 mM HEPES, pH 7.5. After acetone precipitation, equal amounts of outer-membrane proteins fraction (50 μg) were resolved in 10% SDS-PAGE and visualized by InstantBlue Protein Stain. L: protein ladder.

In order to identify any differences in the OMP profiles between the eight *C. malonaticus* strains, their OMPs were extracted and separated using 10% SDS-PAGE (Figure 1). The SDS-PAGE profiles of isolated OMPs from the eight *C. malonaticus* strains revealed the presence of several proteins bands with molecular weights ranging from 150 to 20 kDa. The general protein banding pattern was similar for all *C. malonaticus* strains. However, one dominant band of size ~29 kDa was only present in four *C. malonaticus* strains (5065, 1556, 1827, and 2018), and coincided with their higher invasive ability (Table 1).

**Figure 2:**
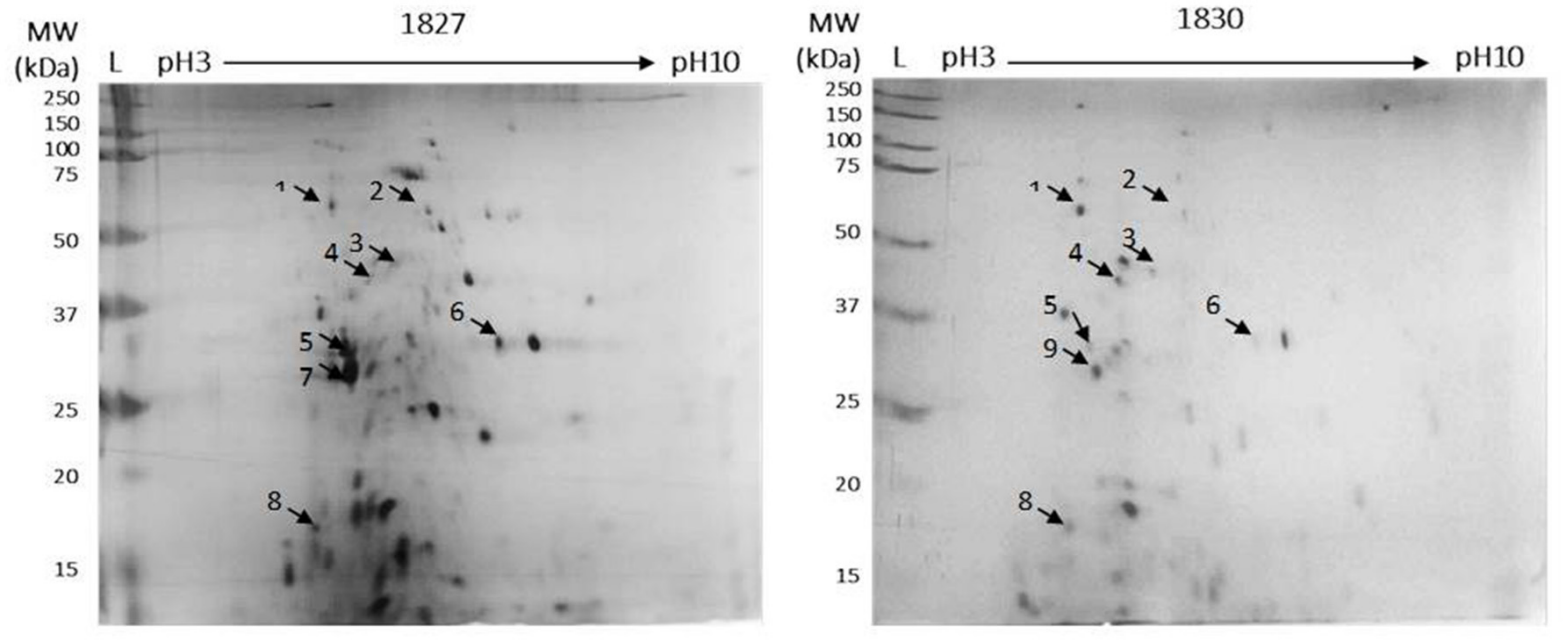
Detection of OMPs for *C. malonaticus* 1827 (highly invasive) and *C. malonaticus* 1830 (low invasive) using 2D-PAGE. For the first-dimension of isoelectric focusing, outer-membrane proteins (50 μg) was loaded onto immobilized pH gradient (IPG) strips with a pH range of 3–10. After subsequent SDS-PAGE in the second dimension, the proteins were fixed and visualized using silver stain. Arrows indicated to selected spot for quantification and identification.

In order to gain a better resolution and to explore the differences in the OMP profiles of the *C. malonaticus* strains, the isolated OMPS from one highly invasive *C. malonaticus* strain (1827) and one low invasive strain (1830) were subjected to further detailed analysis using 2D-PAGE and visualized by silver staining (Figure 2). Strains 1827 and 1830 were chosen as they have the highest and lowest invasion properties, respectively.

Comparison between the two gels identified eight spots, out of 40, that were different between the two strains. These appeared only in the highly invasive *C. malonaticus* strain (1827) gel or their expression level was either higher or lower as compared to the low invasive strain (1830). Eight spots were picked from each gel for identification by MALDI-TOF/TOF. Two out of the eight protein spots (beta-lactamase, and flagellar export ATPase FliL; spots five and six, Figure 2) in the highly invasive *C. malonaticus* strain, 1827, showed over a 2.5 fold increase in intensity compared to the low invasive strain, 1830. In contrast, three protein spots (dihydroorotase, ABC transported substrate-binding protein, and a hypothetical protein) of the low invasive strain showed a significant lower intensity (≥2-fold) compared to the highly invasive strain. Spot 7 in the 1827 strain gel was identified as Flagellin, however the same spot in the 1830 strain gel (spot 9, Figure 2) was identified as NADP-dependent 3-hydroxy acid dehydrogenase. Overall, spot analysis revealed nine proteins that are different between the two strains; seven that differ in their expression level and two specific to each strain (Table 2). Since the predicted size of the protein spot number 7 is not similar to the actual size, this spot was also identified using LC-MS-MS.

**Table 2:** Protein spots identified by MALDI-TOF/TOF mass spectrometer and relevant fold change.

**Spot No.^a^**	**Predicted protein**	**Mascot score^b^**	**Accession no**.	**Calculated pI^c^**	**Mass (kDa)**	**Sequence coverage (%)**	**Matched peptide^d^**	**Protein localization**	**Fold change^e^**	**Anova (p)^f^**
1	Dihydroorotase	92	SFI68846	5.22	45.46	31	11	Cytoplasm	2.1↓	0.017^*^
2	ABC transporter substrate-binding protein	91	BAR60569.1	6.93	60.58	42	18	Periplasmic component	1.0↓	0.899
3	Hypothetical protein	90	WP_036288432	5.35	28.87	46	13	Unknown	1.9↓	0.045^*^
4	Histidinol-phosphate transaminase	89	WP_046605311.1	6.27	39.40	43	11	Cytoplasm	2.8↓	0.002^*^
5	Beta-lactamase SHV-6	92	P96348	7.04	28.25	49	12	Periplasmic component	2.5↑	2.127e-007^*^
6	Flagellar protein export ATPase FliI	79	WP_064564339.1	5.91	48.73	26	8	Outer membrane	2.9↑	5.728e-004^*^
7	Flagellin	91	SAY43719.1	5.11	36.76	22	7	Outer membrane	–	
8	DUF3313 domain-containing protein	88	WP_007946360	8.61	24.29	42	7	Outer membrane	1.1↑	0.637
9	NADP-dependent 3-hydroxy acid dehydrogenase	90	ODU73239	7.08	26.53	40	7	Periplasmic component	–	

a *Number refers to the spot of interest selected from 2D-PAGE, as given in Figure 2*.

b *Protein scores greater than 68 are significant (p < 0.05)*.

c *Isoelectric point of predicted protein calculated from sequence*.

d *Number of peptide matched to predicted protein sequence*.

e *The relevant fold change refers to strain 1827 as compared to strain 1830 generated from the 2D-PAGE spot analysis by Progenesis same spots software. Arrows indicate relative increase and decrease of spot intensity*.

f Anova (p) values of three accumulated 2D-PAGE analyzed by Progenesis same spots software;

* *P < 0.05 was regarded as significant*.

–*Absent protein*.

In order to analyse the full OMP profile, which would enable a comprehensive comparison between the studied strains, 1.5 μg of total OMP extracted from triplicates of the eight *C. malonaticus* strains were analyzed by LC-MS/MS. The majority of the identified proteins were shown to be known OMPs, with the remainder being defined as either uncharacterized proteins or hypothetical proteins with unknown function. The latter's identity was annotated via high sequence similarity using NCBI's protein BLAST database (Table 3). All proteins present within the strains were compared, revealing five flagellar proteins that only appeared in the four highly invasive strains. This included flagellar hook protein (FlgE), flagellar hook-associated protein 1, flagellar hook-associated protein 2 (also known as the flagellar cap protein), flagellin (FliC), and flagellar hook-filament junction protein (FlgL).

**Table 3:** OMP content identified for eight *Cronobacter malonaticus* strains analyzed by LC-MS/MS.

**Protein names**	**Highly invasive**				**Low invasive**				**Notes**	**MW (kDa)**
	**565**	**1558**	**1827**	**2018**	**1830**	**1833**	**1835**	**2020**		
50S ribosomal protein L11		x		x	x	x		x		14.749
Enolase (2-phospho-D-glycerate hydro-lyase, 2-phosphoglycerate dehydratase)		x	x	x	x		x	x		45.55
Flagellar hook protein FlgE	x	x	x	x						42.48
Flagellar hook-associated protein 1	x	x	x	x						58.438
Flagellar hook-associated protein 2 (Flagellar cap protein)	x	x	x	x					Flagellar filament capping protein FliD	49.306
Flagellin (FliC)	x	x	x	x						28.84
LPS-assembly lipoprotein LptE					x					20.17
Outer membrane protein assembly factor BamA					x					89.08
Outer membrane protein assembly factor BamB					x				OmpA family lipoprotein	41.18
Outer membrane protein assembly factor BamD					x					27.57
Uncharacterized protein	x	x	x	x	x			x	Transcriptional regulator	12.178
Uncharacterized protein	x	x	x	x	x	x		x	Hypothetical protein	18.018
Uncharacterized protein				x				x	Hypothetical protein	8.08
Uncharacterized protein	x	x		x	x	x		x	Hypothetical protein	12.37
Uncharacterized protein	x	x	x	x					Flagellar hook-filament junction protein FlgL	34.4
Uncharacterized protein		x	x	x	x			x	Lipoprotein	67.13
Uncharacterized protein		x	x	x	x	x	x	x	Manganese catalase	32.7
Uncharacterized protein		x		x	x	x		x	membrane protein	10.921
Uncharacterized protein		x							MltA-interacting protein MipA	27.555
Uncharacterized protein	x	x	x	x	x	x		x	Murein lipoprotein	8.4
Uncharacterized protein		x							Nucleoside-specific channel-forming protein Tsx precursor	33.09
Uncharacterized protein					x				OmpA family lipoprotein	22.186
Uncharacterized protein	x	x	x	x	x	x	x	x	Outer membrane protein X	18.113
Uncharacterized protein	x	x	x	x	x	x		x	Peptidoglycan-associated outer membrane lipoprotein	18.7
Uncharacterized protein	x	x	x		x		x	x	Phosphoporin PhoE	41.37
Uncharacterized protein	x	x	x	x	x	x	x	x	Porin OmpA	38.35
Uncharacterized protein		x	x	x	x	x		x	YciE/YciF family protein	19.06
UPF0325 protein ESA_03178		x		x	x	x		x	Hypothetical protein	15.06
60 kDa chaperonin (GroEL)	x	x	x	x	x	x	x	x		57.18
10 kDa chaperonin (GroS)	x	x	x	x	x	x	x	x		10.25
50S ribosomal protein (rplL)	x	x	x	x	x	x	x	x		12.23
30S ribosomal protein S3 (rpsC)	x	x	x	x	x	x	x	x		25.82
Elongation factor Tu	x	x	x	x	x	x	x	x		43.42

*x, Indicates protein present*.

Two chaperon proteins [60 kDa chaperonin (GroEL) and 10 kDa chaperonin (GroS)] and two small ribosomal proteins (50S ribosomal protein RplL and 30S ribosomal protein S3 RpsC), and one synthesized protein (Elongation factor Tu) were also identified.

There were differences in the OMP profile of the low invasive strains. The majority of OMPs in *C. malonaticus* 1830 were the OMP assembly factor proteins BamA, BamB, and BamD as well as the LPS-assembly lipoprotein LptE. These were absent from the other three low invasive strain (Table 3).

**Figure 3:**
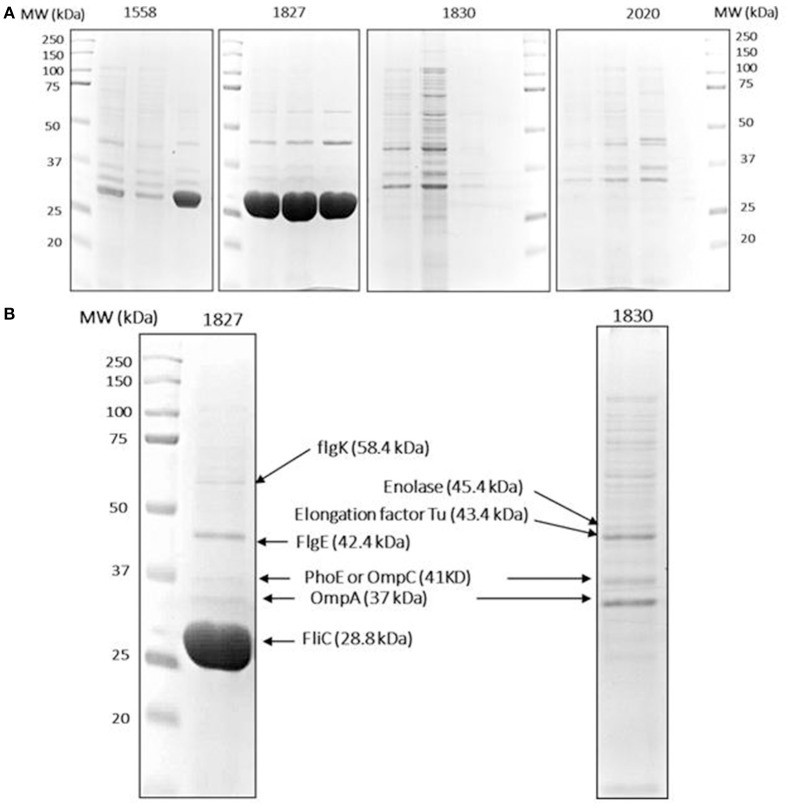
Protein identification data. **(A)** Isolated OMPs of two high (1558, 1872) and two low invasive (1830, 2020) *C. malonaticus* strains from three separate extractions. An equal amount of OMP fraction (50 μg) were resolved in 10% polyacrylamide gel. **(B)** Selected protein bands were extracted from the gels by dehydration and hydration methods. This was followed with trypsin digestion and then directly analyzed by LC-MS/MS in which multiple proteins were identified within a single protein band. Proteins with a high score and sequence coverage are indicated by arrows. L: protein ladder.

Since flagellar-related proteins were shown to be present only in the highly invasive *C. malonaticus* strains, a motility test was performed to determine the motility of the eight strains. This confirmed that the four high invasive strains were motile while the low invasive strains were non-motile (Figure 3). There was some difference in the extent of motility with strain 1558 showing a smaller zone of growth than the other strains.

To further investigate the variation in OMP profile, the OMPs were selectively picked from SDS-PAGE gels and analyzed by LC-MS/MS. For reproducibility, OMPs of the two selected strains (1827 and 1830) along with two further strains (1558 and 2020, high and low invasive strains, respectively) were isolated from three independent extractions and subjected to SDS-PAGE analysis (Figure 3A). The major different band corresponded to size ~29 kDa, along with eight other bands were analyzed by LC-MS/MS for each strain. The mass spectrophotometry results identified no more than two proteins within single bands.

Proteins with a high sequence coverage following peptide mapping are listed in Table 4, along with their molecular weight and biological function. The predicted molecular weight of the proteins is shown corresponding with size of excised band (Figure 3B). The band of size ~29 kDa that was specifically present only in the highly invasive strain (Figure 1) was identified as flagellin (FliC). Flagellar hook-associated protein 1 (FlgK) and flagellar hook protein (FlgE) of band size ~58 and ~43 kDa, respectively, are two further proteins which were identified in the highly invasive strains. Two bands of size ~37 and ~35 kDa were present in both high and low invasive strains. These are listed in Table 4 as uncharacterized proteins, and therefore their sequences were further searched using a protein BLAST search of the NCBI database. The results predicted that the band of size ~37 kDa was phospho-porin (PhoE) belonging to outer membrane porin protein C, while the band of size ~35 kDa was OmpA. Enolase and elongation factor Tu were identified as the ~45 and ~43 kDa bands excised from the SDS-PAGE of the low invasive strains.

**Table 4:** Selected OMPs identified from *C. malonaticus* isolates 1827 or 1830 as analyzed by LC-MS/MS.

**Predicted protein name**	**Sequence coverage (%)**	**Function or family**	**MW (kDa)**
Flagellar hook protein FlgE	48.41	Belongs to the flagella basal body rod proteins family	42.4
Flagellar hook-associated protein 1 FlgK	51.63	Belongs to the flagella basal body rod proteins family	58.4
Enolase (2-phospho-D-glycerate hydro-lyase, 2-phosphoglycerate dehydratase)	41.44	Catalyzes the reversible conversion of 2-phosphoglycerate into phosphoenolpyruvate. It is essential for the degradation of carbohydrates via glycolysis.	45.4
Elongation factor Tu	60.41	This protein promotes the GTP-dependent binding of aminoacyl-tRNA to the A-site of ribosomes during protein biosynthesis	43.4
Uncharacterized protein^a^	54.47	Belongs to the ompA family; Contains OmpA-like domain	37
Flagellin (FliC)	84.89	Flagellin is the subunit protein, which polymerizes to form the filaments of bacterial flagella	28.8
Uncharacterized protein^a^	54.47	Belongs to the Gram-negative porin family; outer membrane porin protein C (phosphoporin PhoE)	41

a *The predicted protein sequence was obtained using protein-BLAST against C. sakazakii; NCBI Reference Sequence: NZ_CP011047.1*.

## Discussion

The most reported *Cronobacter* species in clinical cases are *C. sakazakii* and *C. malonaticus* in infant and adults, respectively. Most reported studies on *Cronobacter* have focused on *C. sakazakii* as it is well known with respect to outbreaks of severe infant infections (necrotizing enterocolitis and meningitis) in neonatal intensive care units. However, the majority of *Cronobacter* infections are in the adult population with various symptoms including urinary tract infections, for which *C. malonaticus* is the more prevalent species isolated (Forsythe et al., 2014; Holy and Forsythe, 2014; Patrick et al., 2014). Although a number of virulence traits have been proposed which may account for the pathogenicity of *C. sakazakii*, there have been fewer studies using *C. malonaticus*.

Cruz-Córdova et al. (2012) published on the induction of pro-inflammatory cytokines in macrophage by the flagella from five species of *Cronobacter*. Their detailed analysis used strains of *C. sakazakii* ST1 and ST4, which are the major pathovars of infant infections. However, they did include the flagellar protein Flic from the *C. malonaticus* type strain (LMG23826^T^, sequence type 7) for comparative purposes. This strain generated more IL-8 release and less TNF-α release from macrophages than the *C. sakazakii* strains, but was not studied further. With respect to surface proteins and cytopathogenicity, Alzahrani et al. (2015) identified 18 outer membrane vesicle associated proteins in a strain of *C. sakazakii* using mass spectrometry. The results indicated that *C. sakazakii* outer membrane vesicles could play a role in pathogenesis by delivering bacterial toxins into host epithelial cells, driving proliferative, and proinflammatory responses. Unfortunately, the study did not extend to *C. malonaticus*.

The accelerating advancement in microbial genomics has helped in the identification of bacterial virulence factors and genes involved in pathogenicity. Nevertheless, differences in pathogenicity can be due to expression differences which are not easily determined using comparative genomic analysis. Thus, the proteomic approach has been applied in this study to compare these eight isolated *Cronobacter malonaticus* strains which were from the same *Cronobacter* sequence type, ST7. This is the major *C. malonaticus* sequence type in the *Cronobacter* PubMLST database (Forsythe et al., 2014). Although *C. malonaticus* strains used in this study are from the same clonal complex (ST7) and therefore have high degree of genomic similarity, they showed differences in their motility and invasive properties (Table 1).

**Figure 4:**
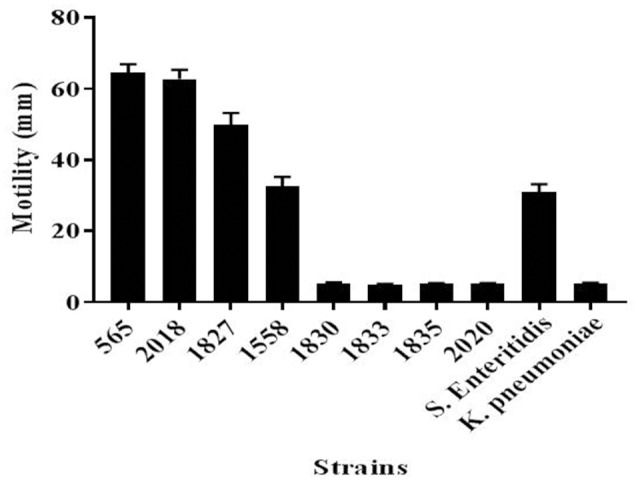
Motility of *C. malonaticus* strains. For each strains, 3 μl of freshly inoculated colonies that were grown in TSB at 37°C for 18 h were inoculated into the center of a TTC-LB agar and incubated overnight at 37°C. The diameter for the motility zone was measured in millimeters. *Salmonella Enteritidis* and *Klebsiella pneumoniae* were used as positive and negative, respectively, motility controls. TSB, Tryptone Soya broth; TTC-LB, Triphenyl-Tetrazolium Chloride-Luria Bertani.

Two-dimensional SDS page coupled with mass spectrophotometry have been used to identify differently expressed virulence related proteins between *C. sakazakii* strains and therefore could be used to study the closely related species *C. malonaticu*s (Du et al., 2015; Ye et al., 2015). In this study, OMPs of eight *C. malonaticus* strains were identified and characterized. The OMPs were identified by two different approaches; mass spectrometry (MALDI-TOF/TOF) of individual OMPs separated by 2D-PAGE profiles (Figure 2), “Bottom up” mass spectrometry (LC-MS/MS) of total OMPs in extraction of eight *C. malonaticus* strains (Table 2) and in bands separated by SDS-PAGE (Figure 4). The OMPs were isolated using Sarkosyl, the most powerful and commonly used approach in OMPs purification of Gram-negative bacteria (Jaradat et al., 2011; Muthiadin et al., 2015; Ferrer-Navarro et al., 2016). The SDS-PAGE profiles for the isolated OMPs of *C. malonaticus* strains (Figure 1) observed in this study were very similar to the OMP patterns detected by other researchers in other *Cronobacter* species and other *Enterobacteriaceae* members (Jaradat et al., 2011; Maiti et al., 2011). Overall, the data shows that the SDS-PAGE profiles for the isolated OMPs are similar in all of *C. malonaticus* strains with the majority of proteins range from ~150 to 30 kDa. However, a single protein band of size ~29 kDa appeared only in four *C. malonaticus* strains (565, 1556, 1827, and 2018). This suggested that the four strains differ in their surface proteins from the other *C. malonaticus*. Based on previous *in vitro* studies these strains were categorized as highly invasive. Furthermore, the strong clonailty within the strains did not give an answer to the observed difference, which made identifying this protein intriguing, as it may be a contributor to the invasion properties of the strains.

This finding was further investigated by identifying the OMPs profile of all eight *C. malonaticus* strains using mass spectrometry (LC-MS/MS). The data shows that the four highly invasive *C. malonaticus* strains share five flagellin-related proteins which include; flagellar hook protein FlgE, flagellar hook-associated protein 1, flagellar hook-associated protein, flagellin, and flagellar hook-filament junction protein FlgL (Table 3). SDS-PAGE revealed that the band of size ~29kDa, which was shared by the four highly invasive *C. malonaticus* strains, was flagellin (FliC) (Figure 4, Table 4). The flagellin (FliC) is a major protein of bacterial flagella, which has been shown to have a crucial role in adhesion and invasion of pathogenic bacteria (Hofstra et al., 1980; Jaradat and Zawistowski, 1998; Haiko and Westerlund-Wikström, 2013). The same protein band of 29kD was previously detected and identified as flagelin in five different *Cronobacter* species using anti-flagella antibody coupled with mass spectrophotometry (Cruz-Córdova et al., 2012). It is also associated with auto-aggregation and biofilm formation (Hartmann et al., 2010; Hoeflinger and Miller, 2017). Flagellar hook-associated protein 1 (FlgK) and flagellar hook protein (FlgE) of band size ~58 and ~43 kDa, respectively, are two further proteins which shows to be specifically expressed in the highly invasive strains (Figure 4 and Table 4).

The comparative analysis of one highly invasive *C. malonaticus* strain (1827) and one low invasive strain (1830) was undertaken using the protein spots from 2D-PAGE. The data shows that two out of the nine protein spots (five and six; Figure 2) in the highly invasive *C. malonaticus* strain 1827 showed a significantly (*p*-value < 0.05) ≥2.5 fold increase compared to the low invasive strain 1830 (Table 2). The identity of these two protein spots was determined by MALDI-TOF/TOF mass spectrometer to be a beta-lactamase (SHV-6), a periplasmic component and flagellar protein export ATPase FliI. In contrast, three protein spots (one, three, and four) of the low invasive strain showed a significant (*p* < 0.05) decrease (≥2-fold) compared to the highly invasive strain (1827). These proteins were also identified as cytoplasmic proteins (dihydroorotase and histidinol-phosphate transaminase), while one protein spot was a hypothetical protein with unknown function, which will be of significant interest in future studies. The presence of other protein including enolase and elongation factor thermo unstable (EF-Tu) is a common finding as they are considered as highly conserved protein that exists on the cell wall of many organisms including gram negative bacteria (Weijland et al., 1992; Diaz-Ramos et al., 2012).

In conclusion, this study has confirmed that flagellar proteins are present only in the highly invasive *C. malonaticus* strains and strongly correlate to their motility. The fact that flagellar proteins are of high importance in the adhesion and invasion of a pathogen supports the theory that the identified flagellar proteins could play a significant role in the invasive abilities of the adult pathovar *C. malonaticus* CC7.

## Author contributions

MA, IA, FB, and AA designed and performed the experiments. MA, IA, and FB analyzed, interpreted the data, and drafted the manuscript. SF provided science direction, contributed to data interpretation, and provided manuscript editing.

### Conflict of interest statement

The authors declare that the research was conducted in the absence of any commercial or financial relationships that could be construed as a potential conflict of interest. The handling Editor declared a past co-authorship with one of the authors SF.

## References

[B1] Aldova E. Hausner O. Postupa R. (1983). Tween esterase activity in *Enterobacter sakazakii*. Z. Bakteriol. Mikrobiol. Hyg. A 256, 103–108. 10.1016/S0174-3031(83)80057-2 6659742

[B2] Almajed F. S. Forsythe S. J. (2016). Cronobacter sakazakii clinical isolates overcome host barriers and evade the immune response. Microb. Pathog. 90, 55–63. 10.1016/j.micpath.2015.11.014 26616163

[B3] Alsonosi A. (2017). Identification of Physiological and Virulence Traits of Clinical Strains of Cronobacter malonaticus. Dissertation/Ph.D. thesis, Nottingham Trent University, Nottingham.

[B4] Alsonosi A. Hariri S. Kajsik M. Orieskova M. Hanulik V. Roderova M.. (2015). The speciation and genotyping of Cronobacter isolates from hospitalised patients. Eur. J. Clin. Microbiol. Infect. Dis. 34, 1979–1988. 10.1007/s10096-015-2440-8 26173692 PMC4565866

[B5] Alzahrani H. Winter J. Boocock D. De Girolamo L. Forsythe S. J. (2015). Characterization of outer membrane vesicles from a neonatal meningitic strain of *Cronobacter sakazakii*. FEMS Microbiol. Lett. 362:fnv085. 10.1093/femsle/fnv085 26023200

[B6] Biering G. Karlsson S. Clark N. C. Jonsdottir K. E. Ludvigsson P. Steingrimsson O. (1989). Three cases of neonatal meningitis caused by *Enterobacter sakazakii* in powdered milk. J. Clin. Microbiol. 27, 2054–2056. 2778070 10.1128/jcm.27.9.2054-2056.1989 PMC267737

[B7] Caubilla-Barron J. Hurrell E. Townsend S. Cheetham P. Loc-Carrillo C. Fayet O.. (2007). Genotypic and phenotypic analysis of *Enterobacter sakazakii* strains from an outbreak resulting in fatalities in a neonatal intensive care unit in France. J. Clin. Microbiol. 45, 3979–3985. 10.1128/JCM.01075-07 17928419 PMC2168550

[B8] Cetinkaya E. Joseph S. Ayhan K. Forsythe S. J. (2013). Comparison of methods for the microbiological identification and profiling of Cronobacter species from ingredients used in the preparation of infant formula. Mol. Cell. Probes 27, 60–64. 10.1016/j.mcp.2012.10.003 23089182

[B9] Cottrell J. S. (2011). Protein identification using MS/MS data. J. Proteomics 74, 1842–1851. 10.1016/j.jprot.2011.05.014 21635977

[B10] Cruz-Córdova A. Rocha-Ramirez L. M. Ochoa S. A. Gonzalez-Pedrajo B. Espinosa N. Eslava C.. (2012). Flagella from five Cronobacter species induce pro-inflammatory cytokines in macrophage derivatives from human monocytes. PLoS ONE 7:E52091. 10.1371/journal.pone.0052091 23284883 PMC3528739

[B11] Diaz-Ramos A. Roig-Borrellas A. Garcia-Melero A. Lopez-Alemany R. (2012). αEnolase, a multifunctional protein: its role on pathophysiological situations. J. Biomed. Biotechnol. 2012:156795. 10.1155/2012/156795 23118496 PMC3479624

[B12] Du X. J. Han R. Li P. Wang S. (2015). Comparative proteomic analysis of *Cronobacter sakazakii* isolates with different virulences. J. Proteomics 128, 344–351. 10.1016/j.jprot.2015.08.013 26327241

[B13] Farmer J. J. Asbury M. A. Hickman F. W. Brenner D. J. The Enterobacteriaceae Study Group (1980). *Enterobacter sakazakii*: a new species of “Enterobacteriaceae” isolated from clinical specimens. Int. J. Syst. Evol. Microbiol. 30, 569–584. 10.1099/00207713-30-3-569

[B14] Ferrer-Navarro M. Balleste-Delpierre C. Vila J. Fabrega A. (2016). Characterization of the outer membrane subproteome of the virulent strain *Salmonella Typhimurium* SL1344. J. Proteomics 146, 141–147. 10.1016/j.jprot.2016.06.032 27373869

[B15] Forsythe S. J. Dickins B. Jolley K. A. (2014). Cronobacter, the emergent bacterial pathogen *Enterobacter sakazakii* comes of age; MLST and whole genome sequence analysis. BMC Genomics 15:1121. 10.1186/1471-2164-15-1121 25515150 PMC4377842

[B16] Friedemann M. (2007). *Enterobacter sakazakii* in food and beverages (other than infant formula and milk powder). Int. J. Food Microbiol. 116, 1–10. 10.1016/j.ijfoodmicro.2006.12.018 17331606

[B17] Giri C. P. Shima K. Tall B. D. Curtis S. Sathyamoorthy V. Hanisch B.. (2012). *Cronobacter* spp. (previously *Enterobacter sakazakii)* invade and translocate across both cultured human intestinal epithelial cells and human brain microvascular endothelial cells. Microb. Pathog. 52, 140–147. 10.1016/j.micpath.2011.10.003 22023990

[B18] Haiko J. Westerlund-Wikström B. (2013). The role of the bacterial flagellum in adhesion and virulence. Biology 2, 1242–1267. 10.3390/biology2041242 24833223 PMC4009794

[B19] Hariri S. Joseph S. Forsythe S. J. (2013). *Cronobacter sakazakii* ST4 strains and neonatal meningitis, United States. Emerg. Infect. Dis. 19, 175–177. 10.3201/eid1901.120649 23260316 PMC3557988

[B20] Hartmann I. Carranza P. Lehner A. Stephan R. Eberl L. Riedel K. (2010). Genes involved in *Cronobacter sakazakii* biofilm formation. Appl. Environ. Microbiol. 76, 2251–2261. 10.1128/AEM.00930-09 20118366 PMC2849266

[B21] Hoeflinger J. L. Miller M. J. (2017). *Cronobacter sakazakii* ATCC 29544 autoaggregation requires FliC flagellation, not motility. Front. Microbiol. 8:301 10.3389/fmicb.2017.00301 28293226 PMC5328975

[B22] Hofstra H. Van Tol J. D. Dankert J. (1980). Cross-reactivity of major outer membrane proteins of Enterobacteriaceae, studied by crossed immunoelectrophoresis. J. Bacteriol. 143, 328–337. 6995435 10.1128/jb.143.1.328-337.1980 PMC294238

[B23] Holy O. Forsythe S. (2014). *Cronobacter* spp. as emerging causes of healthcare-associated infection. J. Hosp. Infect. 86, 169–177. 10.1016/j.jhin.2013.09.011 24332367

[B24] Holy O. Petrzelova J. Hanulik V. Chroma M. Matouskova I. Forsythe S. J. (2014). Epidemiology of *Cronobacter* spp. isolates from patients admitted to the Olomouc University Hospital (Czech Republic). Epidemiol. Mikrobiol. Imunol. 63, 69–72. 24730997

[B25] Hurrell E. Kucerova E. Loughlin M. Caubilla-Barron J. Hilton A. Armstrong R.. (2009). Neonatal enteral feeding tubes as loci for colonisation by members of the Enterobacteriaceae. BMC Infect. Dis. 9:146. 10.1186/1471-2334-9-146 19723318 PMC2749046

[B26] Iversen C. Forsythe S. (2003). Risk profile of *Enterobacter sakazakii*, an emergent pathogen associated with infant milk formula. Trends Food Sci. Technol. 14, 443–454. 10.1016/S0924-2244(03)00155-9

[B27] Iversen C. Mullane N. McCardell B. Tall B. D. Lehner A. Fanning S.. (2008). Cronobacter gen. nov., a new genus to accommodate the biogroups of *Enterobacter sakazakii*, and proposal of *Cronobacter sakazakii* gen. nov., comb. nov., *Cronobacter malonaticus* sp. nov., *Cronobacter turicensis* sp. nov., *Cronobacter muytjensii* sp. nov., *Cronobacter dublinensis* sp. nov., Cronobacter genomospecies 1, and of three subspecies, *Cronobacter dublinensis* subsp. dublinensis subsp. nov., *Cronobacter dublinensis* subsp. lausannensis subsp. nov. and *Cronobacter dublinensis* subsp. lactaridi subsp. nov. Int. J. Syst. Evol. Microbiol. 58(Pt 6), 1442–1447. 10.1099/ijs.0.65577-0 18523192

[B28] Jaradat Z. W. Rashdan A. M. Ababneh Q. O. Jaradat S. A. Bhunia A. K. (2011). Characterization of surface proteins of *Cronobacter muytjensii* using monoclonal antibodies and MALDI-TOF Mass spectrometry. BMC Microbiol. 11:148. 10.1186/1471-2180-11-148 21702985 PMC3224122

[B29] Jaradat Z. W. Zawistowski J. (1998). Antigenically stable 35 kDa outer membrane protein of Salmonella. Food Agric. Immunol. 10, 259–270. 10.1080/09540109809354989

[B30] Joseph S. Forsythe S. J. (2011). Predominance of *Cronobacter sakazakii* sequence type 4 in neonatal infections. Emerg. Infect. Dis. 17, 1713–1715. 10.3201/eid1709.110260 21888801 PMC3322087

[B31] Kandhai M. C. Reij M. W. Gorris L. G. Guillaume-Gentil O. van Schothorst M. (2004). Occurrence of *Enterobacter sakazakii* in food production environments and households. Lancet 363, 39–40. 10.1016/S0140-6736(03)15169-0 14723994

[B32] Kucerova E. Joseph S. Forsythe S. (2011). The Cronobacter genus: ubiquity and diversity. Qual. Assur. Saf. Crops Foods 3, 104–122. 10.1111/j.1757-837X.2011.00104.x

[B33] Laemmli U. K. (1970). Cleavage of structural proteins during the assembly of the head of bacteriophage T4. Nature 227, 680–685. 10.1038/227680a0 5432063

[B34] Lai K. K. (2001). *Enterobacter sakazakii* infections among neonates, infants, children, and adults. Case reports and a review of the literature. Medicine 80, 113–122. 10.1097/00005792-200103000-00004 11307587

[B35] Maiti B. Shetty M. Shekar M. Karunasagar I. (2011). Recombinant outer membrane protein A (OmpA) of *Edwardsiella tarda*, a potential vaccine candidate for fish, common carp. Microbiol. Res. 167, 1–7. 10.1016/j.micres.2011.02.002 21482086

[B36] Miled-Bennour R. Ells T. C. Pagotto F. J. Farber J. M. Kerouanton A. Meheut T.. (2010). Genotypic and phenotypic characterisation of a collection of Cronobacter (*Enterobacter sakazakii*) isolates. Int. J. Food Microbiol. 139, 116–125. 10.1016/j.ijfoodmicro.2010.01.045 20181403

[B37] Muthiadin C. Natsir R. Agus R. Nasrum M. Dwiyanti R. Sabir M. (2015). Identification and characterization of antigenic 36 Kda Outer Membrane Protein (OMP) of *Salmonella enterica* serovar from Makassar, South Sulawesi, Indonesia. Am. J. Biomed. Res. 3, 9–12. 10.12691/ajbr-3-1-3

[B38] Nirmalan N. Sims P. F. Hyde J. E. (2004). Quantitative proteomics of the human malaria parasite *Plasmodium falciparum* and its application to studies of development and inhibition. Mol. Microbiol. 52, 1187–1199. 10.1111/j.1365-2958.2004.04049.x 15130134

[B39] Patrick M. E. Mahon B. E. Greene S. A. Rounds J. Cronquist A. Wymore K. (2014). Incidence of *Cronobacter* spp. infections, United States, 2003-2009. Emerg. Infect. Dis. 20, 1520–1523. 10.3201/eid2009.140545 25148394 PMC4178417

[B40] Sonbol H. Joseph S. McAuley C. M. Craven H. M. Forsythe S. J. (2013). Multilocus sequence typing of *Cronobacter* spp. from powdered infant formula and milk powder production factories. Int. Dairy J. 30, 1–7. 10.1016/j.idairyj.2012.11.004

[B41] Stoscheck C. M. (1990). Quantitation of protein. Methods Enzymol. 182, 50–68. 10.1016/0076-6879(90)82008-P 2314256

[B42] Townsend S. Hurrell E. Forsythe S. (2008). Virulence studies of *Enterobacter sakazakii* isolates associated with a neonatal intensive care unit outbreak. BMC Microbiol. 8:64. 10.1186/1471-2180-8-64 18423002 PMC2386127

[B43] Townsend S. M. Hurrell E. Gonzalez-Gomez I. Lowe J. Frye J. G. Forsythe S.. (2007). *Enterobacter sakazakii* invades brain capillary endothelial cells, persists in human macrophages influencing cytokine secretion and induces severe brain pathology in the neonatal rat. Microbiology 153(Pt 10), 3538–3547. 10.1099/mic.0.2007/009316-0 17906151

[B44] Weijland A. Harmark K. Cool R. H. Anborgh P. H. Parmeggiani A. (1992). Elongation factor Tu: a molecular switch in protein biosynthesis. Mol Microbiol. 6, 683–688. 10.1111/j.1365-2958.1992.tb01516.x 1573997

[B45] Ye Y. Gao J. Jiao R. Li H. Wu Q. Zhang J.. (2015). The membrane proteins involved in virulence of *Cronobacter sakazakii* virulent G362 and attenuated L3101 isolates. Front. Microbiol. 6:1238. 10.3389/fmicb.2015.01238 26617581 PMC4637405

